# Dataset overlap density analysis

**DOI:** 10.1186/1758-2946-5-S1-O14

**Published:** 2013-03-22

**Authors:** Andreas H Göller

**Affiliations:** 1 Bayer Schering Pharma AG, Computational Chemistry, Wuppertal, Germany

## 

The need to compare compound datasets arises from various scenarios, like mergers, library extension programs, gap analysis, combinatorial library design, or estimation of QSAR model applicability domains. Whereas it is relatively easy to find identical compounds in two datasets, the quantification of the overlap is not straightforward. The various approaches described include pairwise nearest neighbor comparisons, clustering and mixed cluster statistics, or binning of e.g. rule-of-five property space distributions. The BCUT methodology creates a binned N-dimensional space and allows to assess the amount of mixed cells. ChemGPS creates a PCA reference projection based on drug-like and satellite molecules in property space to classify new compounds.

But is it possible and also plausible to quantify the overlap of two datasets in a single interpretable number?

PCA projection models with the World Drug Index as drug-like reference space were created based on MACCS, ECFP4, estate or Lipinsky-like physchem descriptors. Compounds from the commercial vendor i-research library, ZINC, ChEMBL and a current screening subset from PubChem were projected onto the WDI maps.

The dataset overlap density index DOD is calculated from the summations over the occupancies of each N-dimensional "volume" element occupied by both datasets, divided by all such elements populated by at least one dataset. The index provides a measure of the overlap of two sets.

It is shown that the number of principal components needed to describe at least 75% of the information content of the descriptor greatly varies and that a projection in 2 dimensions is not adequate. Such N-dimensional projections are extremely sparse (about 1043 elements for WDI and MACCS descriptor) and crowded only in small regions of the spanned N-dimensional space.

The approach is universal to any descriptor. It can be extended to a DOD vector based on different descriptor types each describes different characteristics of the encoded molecules. The box element graining can be easily adjusted as needed for a particular application. based on needs. It allows to quantify local gaps or overlaps. Proprietary datasets can be compared just by the first N principal component values without even seeing the descriptors behind.

